# Ultrasound-Assisted Extraction of Arabinogalactan and Dihydroquercetin Simultaneously from *Larix gmelinii* as a Pretreatment for Pulping and Papermaking

**DOI:** 10.1371/journal.pone.0114105

**Published:** 2014-12-02

**Authors:** Chunhui Ma, Lei Yang, Wei Li, Jinquan Yue, Jian Li, Yuangang Zu

**Affiliations:** 1 College of Material Science and Engineering, Northeast Forestry University, 150040, Harbin, China; 2 Key Laboratory of Forest Plant Ecology, Ministry of Education, Northeast Forestry University, 150040, Harbin, China; College of Agricultural Sciences, United States of America

## Abstract

An ultrasound-assisted extraction (UAE) method using ethanol was applied for extracting arabinogalactan (AG) and dihydroquercetin (DHQ) simultaneously from larch wood, as a pretreatment for pulping and papermaking. The extraction parameters were optimized by a Box-Behnken experimental design with the yields of AG and DHQ as the response values. Under optimum conditions (three extractions, each using 40% ethanol, for 50 min, 200 W ultrasound power and 1∶18 solid-liquid ratio), the yields of AG and DHQ were 183.4 and 36.76 mg/g, respectively. After UAE pretreated, the wood chips were used for Kraft pulping (KP) and high boiling solvent pulping (HBSP). The pulping yield after pretreatment was higher than that of untreated (the pulping yields of untreated HBSP and KP were 42.37% and 39.60%, and the pulping yields of HBSP and KP after UAE-pretreated were 44.23% and 41.50% respectively), as indicated by a lower kappa number (77.91 and 27.30 for untreated HBSP and KP; 77.01 and 26.83 for UAE-pretreated HBSP and KP). Furthermore, the characteristics of paper produced from pretreated wood chips were superior to those from the untreated chips: the basis weight was lower (85.67 and 82.48 g·cm^−2^ for paper from untreated KP and HBSP; 79.94 and 80.25 g·cm^−2^ for paper from UAE-pretreated KP and HBSP), and the tensile strengths, tearing strengths, bursting strengths, and folding strengths were higher than these of paper after UAE-pretreated, respectively.

## Introduction

The larch is a conifer of the *Larix* genus, from the Pinaceae family [1], which is native to the cooler temperate climate of the northern hemisphere, especially in the boreal forests of Russia and Canada [2]. *Larix gmelinii* (syn. L. *dahurica*) is a species, unique to the northeastern forests of China, a temperate coniferous broad-leaved mixed forest area, accounting for the largest proportion of forest trees [3]. The chemical composition of *Larix gmelinii* comprises cellulose, lignins, hemicellulose (arabinogalactan), ash and extractable compounds (resins and dihydroquercetin). It is also an important source of pulp wood for papermaking.

However, arabinogalactan (AG) and dihydroquercetin (DHQ) cannot be ignored in the pulping process. A large amount of alkali is consumed and the temperature has to rise slowly, because of AG in the raw material, such as the Kraft pulping process. Because the mechanism of lignin removal relies on the degradation of hemicelluloses, the degradation of AG occurs at the heating stage under alkaline conditions [4]. If AG could be extracted before the pulping operation, this would not only reduce the amount of alkali used, but also shorten the heating time. Meanwhile, DHQ, as well as tannins and polyphenols, is also present in larch wood. The higher degree of condensation of the phenolic hydroxyl group causes difficulty in the pulping process [5]. If the compounds containing phenolic hydroxyl groups could be extracted before pulping operation, this could facilitate the bleaching process for pulping.

AG and DHQ are active compounds in larch wood and have usually been wasted in the pulping process, but they also have many pharmacological effects.

AG is a biopolymer consisting of arabinose and galactose monosaccharides, which has a galactan core connected to D-galactopyranose by β-1,3-O-glucoside links [6]. AG is present in the larch woody tissue at a level of 15-30% [7]. Larch AG is a highly branched polysaccharide consisting of β-D-galactopyranose, α-L-arabinofuranose and β-L-arabinopyranose residues [8]. AG was generally recognized as safe by the FDA, USA in 1974. It is well accepted by consumers as a dietary supplement [9] and food ingredient, because of its water-soluble and non-viscous properties [10]. Moreover, many pharmacological effects of AG have been reported, such as anti-inflammatory [6], gastro-protective [6], membranetropic [11], and immune-modulating activities [12]. Additionally, AG can be converted into more valuable products through a sugar platform [8]. Therefore, AG has been recognized as a multi-purpose natural product with great economic potential and environmental value, which has attracted increasing attention by researchers. AG is commonly extracted with water and precipitated using the ethanol method, which needs a higher-volume fraction of ethanol solution to precipitate AG thoroughly [13].

**Figure 1:**
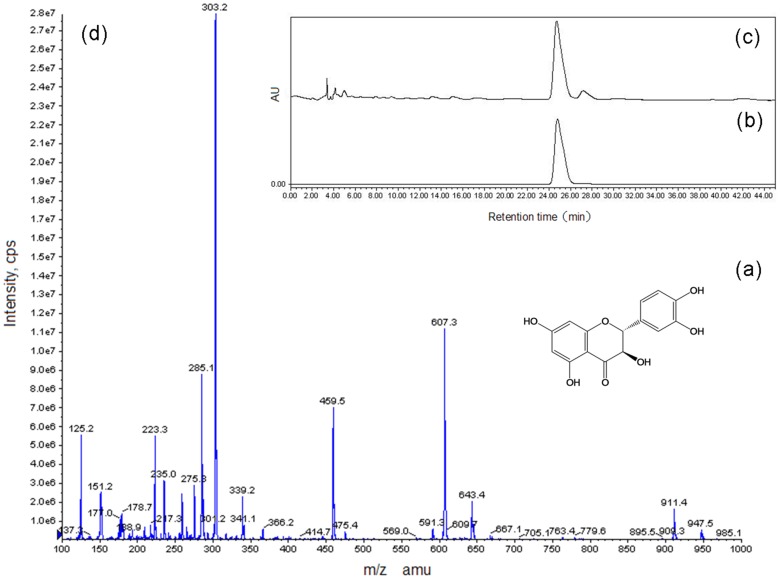
HPLC-UV and LC-ESI-MS analysis of DHQ. (a) Chemical structure of DHQ. (b) HPLC-UV chromatogram of DHQ standard. (c) HPLC-UV chromatogram of Larch wood sample. (d) LC-ESI-MS chromatogram of DHQ.

DHQ also known as taxifolin (2-(3,4-dihydroxyphenyl)-2,3-dihydro-3,5,7- trihydroxy-4H-benzopyran-4-one) and vitamin P [14], and the structure is shown in Figure 1a. DHQ have many biological activities, including the inhibition or activation of a variety of enzymes, resulting in different physiological effects; the protection of cells against oxidative stress, attributed to its antioxidant properties [15]; and also anti-radiation [16], anti-viral [17], anti-tumor [18] and scavenging free radical [19] properties because of its rich content of phenolic hydroxyl groups. The antioxidant properties of DHQ can be comparable or superior to many synthetic or natural antioxidants. Moreover, it is not toxic to the fetus, teratogenic, mutagenic or allergenic. DHQ is commonly extracted from wood using a polar solvent, such as methanol or ethanol, or by Soxhlet, reflux, ultrasonic or accelerated solvent extraction [20]. Supercritical fluid extraction (CO_2_) can be applied, but a modifier such as methanol is then required [21]. The use of enzymatic water extraction of DHQ from wood material has been proposed by Wang et al. [22], but the increasing cost of enzyme disposal has created a growing demand for better and cheaper extraction methods for DHQ.

Recently, much more attention has been given to the application of ultrasound-assisted extraction (UAE) in the fields of analytical chemistry and sample preparation (i.e. digestion, extraction and dissolution) [23]. Compared with conventional solvent extraction, the use of ultrasound makes the extraction of valuable compounds more efficient by using shorter time frames and lower extraction temperatures [24]. The possible benefits of ultrasound in extraction are the intensification of mass transfer, cell disruption, improved penetration and capillary effects [25]. Ultrasound is currently used to extract such pharmacologically active compounds as polysaccharides [13], [25], flavonoids [21], [22] and isoflavonoids [24], alkaloids [26], lignans [27], anthraquinone [28], carnosic and rosmarinic acids [29], steroids and triterpenoids [30] from plant materials. However, there are few reports of applying UAE for extracting two or more kinds of natural products simultaneously from wood or plant materials.

The primary aim of the present study is to optimize the UAE conditions for isolating AG and DHQ simultaneously from larch wood using a lower-volume fraction of ethanol solution as a pretreatment for the pulping and papermaking processes. AG will then be precipitated using a higher-volume fraction of ethanol solution so that AG and DHQ can be separated. The wood chips pretreated by UAE will be used as the raw material for pulping. Any changes in the pulping characteristics and physical properties of the paper will be determined. Compared with the conventional extraction methods, the invocation of this study is not only obtained the active compounds, AG and DHQ in one step, also improve the quality of pulping and papermaking which made from the pretreatment larch wood after extracting AG and DHQ.

## Materials and Methods

### Wood materials

*Larix gmelinii* wood chips (average size 25×20×6 mm) were purchased from Greater Khingan Mountains (Heilongjiang, China) and identified by Academician Jian Li from the College of Material Science and Engineering, Northeast Forestry University, Harbin, China. The moisture content of the wood chips was 5.9% after drying in a shaded and ventilated place, with no further grinding processing before use.

For determining the composition, 100.0 g wood flour after crushing was precisely weighed. Larch wood contains cellulose (52.4%±0.6%), lignin (28.2%±0.3%), benzene alcohol extractives (5.2%±0.1%), arabinogalactan (22.3%±0.5% using Soxhlet extraction 12 h with water) and dihydroquercetin (6.3%±0.2% using Soxhlet extraction 12 h with 40% ethanol).

### Chemical materials

Dihydroquercetin (DHQ) standards (1257117-20607065, 98% purity) were purchased from the National Institute for the Control of Pharmaceutical and Biological Products (Beijing, China). Deionized water was purified using a Milli-Q Water Purification system (Millipore, Billerica, MA, USA). Acetonitrile and acetic acid of HPLC grade were purchased from J&K Chemical Ltd. (shanghai, China). The rest of the solvents and chemicals used in this study were of analytical grade and purchased from Beijing Chemical Reagents Co. (Beijing, China). All solutions prepared for HPLC were filtered through 0.45-µm membranes (GuangFu Chemical Reagents Co., Tianjin, China) before use.

### HPLC-UV quantitative analysis method of DHQ

The HPLC-UV system consisted of a Waters 717 automatic sample handling system composed of an HPLC system equipped with 1525 Bin pump, 717 automatic column temperature control box and 2487 UV-detector (Waters, Milford, MA, USA). Chromatographic separation was performed on a HiQ sil-C18 reversed-phase column (4.6 mm×250 mm, 5 µm, KYA TECH Corp., Tokyo, Japan) for the determination of DHQ.

For HPLC-UV analysis, acetonitrile-water-acetic acid (18∶82∶0.1, v/v/v) was used as the mobile phase at a flow rate of 1.0 ml/min with a 10 µL injection volume and 25°C column temperature. The absorbance was measured at a wavelength of 294 nm for the detection of DHQ with a run time of 30 min. The retention time of DHQ is 24 min. The corresponding calibration curve for DHQ is Y = 3.1755×10^7^ *X*+2.5993×10^4^ (r = 0.9999). A high degree of linearity was found for DHQ over the range 0.0312–0.5000 mg/ml. The HPLC-UV chromatograms of the DHQ standard and a larch wood sample are shown in Figure 1(b) and 1(c).

### LC-ESI-MS qualitative analysis method of DHQ

The HPLC-ESI-MS system consisted of an Agilent 1100 series HPLC system equipped with a G1312A Bin pump, a G1379A Degasser (Agilent, San Jose, CA, USA) and a G1316A automatic column temperature control box. Chromatographic separation was performed on a HiQ sil-C18 reversed-phase column (4.6 mm×250 mm, 5 µm, KYA TECH). An API3000 Triple tandem quadrupole mass spectrometer with a Turbolon-Spray interface from Applied Biosystems (Foster City, CA, USA) was operated in the positive electrospray ionization (ESI+) source mode. All mass spectra were acquired in multiple reaction monitoring transitions.

For HPLC-ESI-MS analysis, acetonitrile-water-acetic acid (18∶82∶0.1, v/v/v) was used at a flow rate of 1.0 ml/min with a run time of 65 min. The injection volume was 10 µl and the column temperature was maintained at 25°C. The ion source was operated at a temperature of 250°C. The nebulizing gas, curtain gas and collision gas were set at 12, 10 and 6 a.u., respectively. The ion spray voltage was 5500 V. The entrance potential and focusing potential were set at 10 and 400 V, respectively. Analyst software (version 1.4, Ab Sciex, Framingham, MA, USA) installed on a Dell computer was used for data acquisition and processing. The LC-ESI-MS chromatogram of DHQ is shown in Figure 1(d).

### UAE method for extracting AG and DHQ as pretreatment

For the ultrasonic-assisted extraction (UAE) experiments, an ultrasonic bath was used as an ultrasonic source. The bath (KQ-250DB, Kunshan Ultrasonic Co. Ltd., China) was a open rectangular container (23.5×13.3×10.2 cm), to which 50 kHz transducers were annealed at the bottom. The bath power rating is 250 W, and the power is divided into 5 file, are 50, 100, 150, 200 and 250 W, respectively.

The extraction experiment was performed by adding 30.0 g of wood materials into different extraction solvent in a 500 mL glass flask. The flask was then partially immersed into the ultrasonic bath, which contains 2.5 L of water. The water in the ultrasonic bath is circulated and regulated at the desired temperature to maintain the water temperature at a constant value (25°C) and prevent it from being influenced by the ultrasonic exposure. After ultrasonic procedure, the extracts are then cooled down to the room temperature, and filtrated through a 0.45 µm filter prior to HPLC analysis.

### Effect of the volume fraction of ethanol for extracting AG and DHQ as pretreatment method

Polysaccharides are commonly extracted with water and precipitated using a higher-volume fraction of ethanol solution. To extract AG and DHQ from larch wood simultaneously, the volume fraction of ethanol as the extraction solvent was investigated. First, AG and DHQ were extracted simultaneously using UAE with a lower-volume fraction of ethanol solution, and then AG was precipitated using a higher-volume fraction of ethanol solution. The precipitated effect was shown in Table 1 (the original data was in File S1). Finally, AG and DHQ were separated using centrifugation at 10000 r/min.

**Table 1:** Effect of ethanol volume fraction on precipitation yield of AG.

Volume fraction of ethanol	Rotated speed (r/min)	precipitation yield of AG (mg/g)	Precipitation phenomenon of AG
20%	10000	6.28±0.34	No obvious precipitation
30%	10000	11.86±0.24	No obvious precipitation
40%	10000	25.37±0.75	No obvious precipitation
50%	10000	40.35±0.41	Milky turbidity
60%	10000	58.48±0.79	Milky turbidity
70%	10000	141.60±1.47	White flocculent precipitation
80%	10000	168.79±1.74	White flocculent precipitation
90%	10000	172.87±1.76	White flocculent precipitation

### Single-factor test method

To study the effect of the solid-liquid ratio, soaking time, ultrasound extraction time and ultrasound power on the extraction yields of AG and DHQ, the single-factor test was used three times and the average value was recorded.

### Optimization test by Response Surface Methodology (RSM)

To further investigate the interaction between the factors in the process of UAE, we optimized the operating conditions using Response Surface Methodology (RSM) and the Box-Behnken software for data processing. The bounds of the factors were 40–60 min for extraction time, 150–250 W for ultrasound power and 1∶16–1∶20 for the solid-liquid ratio. As the volume fraction of ethanol and soaking time are not parameters in the process of UAE, it cannot be optimized by the software. The volume fraction of ethanol was 40% and the soaking time was 8.0 h. Specific protocols for experimental conditions and response values are shown in Table 2 (the original data, File S1).

**Table 2:** Experimental design matrix to screen important variables for extraction yields of AG and DHQ.

Run	Factor *A*	Factor *B*	Factor *C*	Response *Y_1_*	Response *Y_2_*
	Extraction time (min)	Ultrasound power (W)	Solid-liquid ratio (g/mL)	Extraction yield of DHQ (mg/g)	Extraction yield of AG (mg/g)
1	40	250	1∶18	36.1	163.4
2	50	150	1∶16	34.2	154.3
3	50	250	1∶20	36.9	183.2
4	60	200	1∶20	37.1	185.4
5	40	200	1∶20	36.3	169.5
6	60	250	1∶18	36.6	179.7
7	40	200	1∶16	36.2	161.7
8	50	200	1∶18	36.7	183.4
9	50	200	1∶18	36.8	183.2
10	50	150	1∶20	35.6	158.3
11	60	150	1∶18	35.1	160.2
12	40	150	1∶18	34.5	156.8
13	50	200	1∶18	36.7	183.8
14	50	200	1∶18	36.9	183.5
15	50	200	1∶18	36.7	183.1
16	60	200	1∶16	36.0	163.3
17	50	250	1∶16	36.5	176.7

### Effect of the extraction cycles on the yields and extraction efficiency of AG and DHQ

The number of extraction cycles is a crucial factor in the extraction process; the more extraction cycles, the higher the dissolution efficiency of the active ingredient. However, there is difficulty in the consumption and recycling of extraction solvent as the number of extraction cycles increases. Four extraction cycles were used and the total extraction efficiency from them was defined as 100%.

### Reference extraction methods

The conventional extraction methods for AG are reflux extraction with water and soaking or stirring extraction with water, and for DHQ, reflux extraction with hot water or ethanol solutions, and soaking or stirring extraction with ethanol. The extraction yields of AG and DHQ from the various extraction methods were compared with the optimal conditions of UAE-pretreated predicted by RSM: 40% ethanol solution, 50 min ultrasound extraction at 200 W and 1∶18 solid-liquid ratio).

### Pulping method and the physical properties of paper

To investigate the physical properties of wood pulp and paper, two kinds of pulping process were studied. One was the traditional Kraft pulping process, and the other, currently of high interest to researchers, was the high boiling solvent pulping process.

#### Kraft pulping process (KP)

UAE-pretreated wood chips were mixed using 20% causticity, 25% sulfidity (sodium sulfide concentration), at a solid-liquid ratio of 1∶3.5. The mixture was put into a ZQS1 electric cooking pot (Machinery Factory of Shanxi University of Science and Technology, Shanxi, China) then heated up to 140°C at a pressure of 0.4 KPa, with a small release steam for 3 min to eliminate any false pressure. The mixture was then heated up to a maximum temperature of 170°C, at a pressure of 0.8 KPa with thermal retardation for 60 min, then a large release of steam was made until the pressure changed to zero. After the reaction, solids and liquids were separated and the mixture was then washed. After washing, the pulp was suitable for further processing into paper using a QJ1-B-II sheet molding machine (Machinery Factory of Shanxi University of Science and Technology, Shanxi, China).

#### High boiling solvent pulping process (HBSP)

UAE-pretreated (the optimal conditions predicted by RSM: 40% ethanol solution, 50 min ultrasound extraction at 200 W and 1∶18 solid-liquid ratio) wood chips were mixed with a volume fraction of 80% ethanol with 10% acetic acid as the catalyst. The reaction temperature and pressure were 200°C and 1.3 MPa, respectively. The reaction was performed using a T25FYX autoclave and FDK autoclave controller (Dalian Industry Autoclave Vessel Manufacturing Co. Ltd., Liaoning, China). The reaction continued for 6 h followed by solid-liquid separation. After washing, the pulp was suitable for further processing into paper using a QJ1-B-II sheet molding machine (Machinery Factory of Shanxi University of Science and Technology, Shanxi, China).

#### Determination of the physical properties of paper

To compare the physical properties of untreated pulp and its resulting paper and UAE-pretreated (the optimal conditions predicted by RSM: 40% ethanol solution, 50 min ultrasound extraction at 200 W and 1∶18 solid-liquid ratio) pulp and its resulting paper, the parameters measured were the kappa number (according to GB/T1546-1989), basis weight, apparent density, tensile strength (according to GB/T453-1989), tearing strength (according to GB/T455.1–1989), bursting strength (according to GB/T454-1989) and folding strength (according to GB/T454-1989). The instruments used in the tests were produced by the Changchun Small Testing Machine Co. Ltd. (Jilin, China) and consisted of a ZUS-4 paper thickness tester. a ZDNP-1 electronic paper bursting tester, a ZLD-300 electronic paper tensile tester, a ZSE-1000 paper tear tester and a YQ-Z-31 MIT folding endurance tester.

### Statistical analysis method

Results were expressed as mean values ±SD (n = 3). Statistical significance was determined using Microsoft Excel statistical fractions. The data of optimal experiment were analyzed using RSM software design 7.0. Differences at P<0.05 were considered to be significant.

## Results and Discussion

### Factors affecting UAE

#### Extraction solvent

As is well known, AG dissolves in water and its solubility decreases as the volume fraction of ethanol solution increases. DHQ can dissolve in any volume fraction of ethanol solution. To extract AG and DHQ simultaneously, we investigated the volume fraction of ethanol used as an extraction solvent and also as a precipitation solvent. First, AG and DHQ were extracted using UAE with lower volume fraction of ethanol solution, then AG was precipitated by adding anhydrous ethanol, after being centrifuged at 10000 r/min. The optimal volume fraction of ethanol solution for AG precipitation was selected through calculating the extraction yield of AG. From Table 1 (the original data, File S1), when the volume fraction of ethanol in the solution was less than 40% (40% ethanol as the precipitation solvent, the AG yield after being centrifuged was 25.37±0.75 mg/g), there was no obvious precipitation of AG. With more than 50% ethanol in the solution, the yield of AG showed an upward trend with the AG solution showing a milky turbidity (the AG yield was 40.35±0.41 mg/g when 50% ethanol as the precipitation solvent, and 58.48±0.79 mg/g when 60% ethanol as the precipitation solvent). When the volume fraction of ethanol was more than 70%, a white flocculent precipitation occurred within a short time and the yield of AG also showed an upward trend (the AG yield was 141.59±1.47 mg/g when 70% ethanol as the precipitation solvent). When the volume fraction of ethanol was more than 80% (the AG yield was 168.78±1.74 mg/g when 80% ethanol as the precipitation solvent), the upward trend of AG yield was less pronounced (the AG yield was 172.87±1.76 mg/g when 90% ethanol as the precipitation solvent), so an 80% ethanol solution was selected as the precipitation solvent (the precipitation mass by 80% ethanol reached 97.6% of the precipitation mass by 90% ethanol).

**Figure 2:**
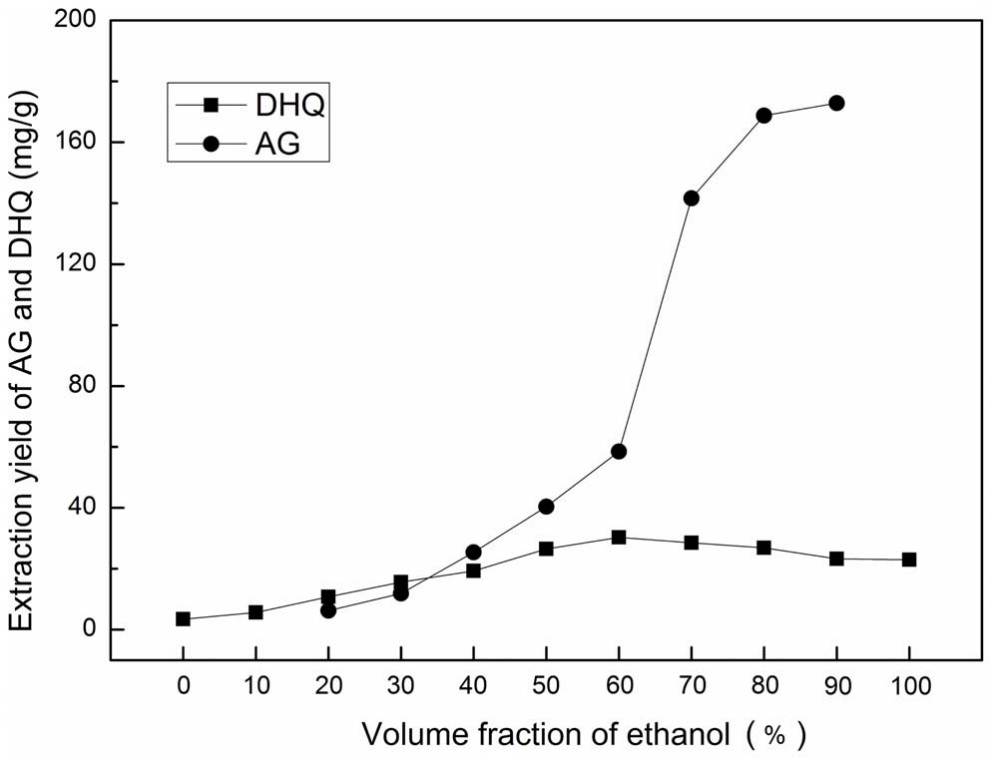
Effect of volume fraction of ethanol on the extraction yields of AG and DHQ.

As can be seen in Figure 2 (the original data, File S1), when the volume fraction of ethanol solution increased, the yield of DHQ increased, with a maximum value when the volume fraction of ethanol solution was 60%. However, when the volume fraction of ethanol was 40%, the precipitation of AG did not occur rapidly, and the yield of DHQ showed little change from the maximum value. Therefore, to extract AG and DHQ simultaneously, a 40% ethanol solution as the extraction solvent and an 80% ethanol solution as the precipitation solvent were selected.

#### Solid-liquid ratio

**Figure 3:**
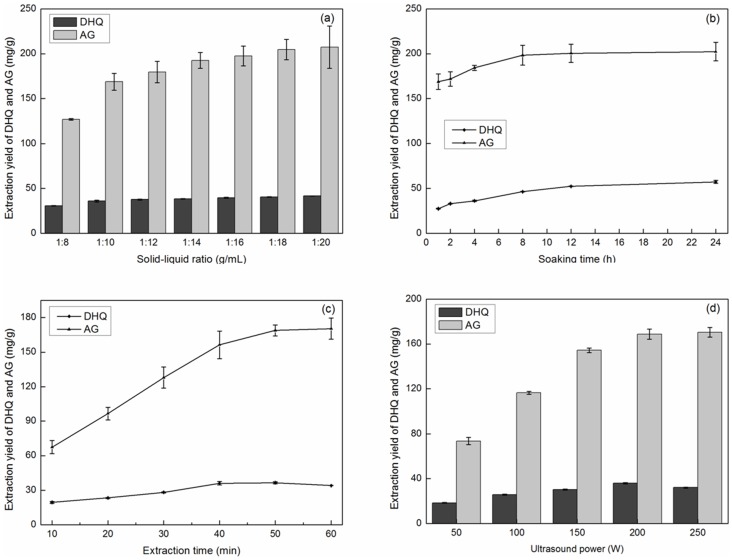
Effect of single factors on the extraction yields of AG and DHQ. (a) Effect of solid-liquid ratio on the extraction yields of AG and DHQ. (b) Effect of soaking time on the extraction yields of AG and DHQ. (c) Effect of extraction time on the extraction yields of AG and DHQ. (d) Effect of ultrasound power on the extraction yields of AG and DHQ.

A high solvent content may cause complex procedures and unnecessary wastage and increase the energy consumption during recycling, while a low solvent content may lead to incomplete extraction. To evaluate the effect of the solid-liquid ratio, 30.0 g of dried wood material was mixed with 40% ethanol solution as the extraction solvent, and then soaked for 8 h, the suspension was extracted 50 min by UAE at the power of 200 W. A series of extractions were carried out using different solid-liquid ratios (1∶8–1∶20 g·ml^−1^). Figure 3a (the original data, File S1) indicates clearly that the extraction yield for AG increased with the increase in solvent volume, but at more than 1∶18 (the yields of AG was 204.66±11.40 with 1∶18 solid-liquid ratios), it was not significantly influenced by a further increase in the amount of solvent. The extraction yield of DHQ did not increase significantly when the solid-liquid ratios were more than 1∶12 (the extraction yields of DHQ was 37.71±0.63 mg/g with 1∶12 solid-liquid ratios). Therefore, considering the yields of AG and DHQ, a range of solid-liquid ratios of 1∶16–1∶20 was selected and used in the further studies.

#### Soaking time

The role of infiltration in the extraction process is to make sure that the raw materials are fully soaked without consuming energy; this can help the dissolution of small molecules into the solution. The longer the time of infiltration, the better the soaking, but a long infiltration time may lead to moldy materials (if the solvent is water) and a long extraction cycle may lead to low extraction efficiency. In the present study, 30.0 g dried wood chips were soaked with 40% ethanol for 0, 1, 2, 4, 8, 12 or 24 h. The solid-liquid ratio was 1∶18, the suspension was extracted 50 min by UAE at the power of 200 W, and the effect of soaking time was studied. Figure 3b (the original data, File S1) shows that the yields of AG and DHQ increased as the soaking time increased up to 8 h then remained almost unchanged after 8 h (the extraction yields of AG and DHQ were 198.36±11.13 and 46.37±0.33 mg/g, respectively). To save time from a full infiltration, the soaking time used was 8 h for the further experiments.

#### Extraction dynamics of DHQ

To investigate the effect of extraction time on the yields of AG and DHQ using UAE, the process was performed in an ultrasound unit. 30.0 g of dried sample was mixed with 40% ethanol soaked for 8 h, and then extracted for 60 min. The power of UAE was 200 W with a solid-liquid ratio of 1∶18, and the yields of AG and DHQ were tested every 10 min. Figure 3c (the original data, File S1) shows that as the extraction time increased, the yields initially increased. Then after a UAE process for 40 min (the extraction yields of AG and DHQ were 156.32±11.94 and 36.00±1.53 mg/g) and 50 min (the extraction yields of AG and DHQ were 168.79±4.81 and 36.52±0.96 mg/g), respectively, the yields of DHQ and AG were almost unchanged. Therefore, a 40-60-min ultrasound treatment was selected as the optimal condition for extracting AG and DHQ.

#### Energy intensity of UAE

To examine the effect of ultrasound power on the extraction yields of AG and DHQ, 30.0 g of dried sample was mixed with 40% ethanol (the solid-liquid ratio was 1∶18) soaked for 8 h, and then were carried out with a constant ultrasonic treatment time of 50 min at 50, 100, 150, 200 and 250 W, respectively. Figure 3d (the original data, File S1) indicates that the average extraction yields of AG and DHQ were significantly increased with the ultrasound power increasing. However, when the ultrasound power levels greater than 200 W (the extraction yields of AG and DHQ were 168.79±4.47 and 35.96±0.63 mg/g at 200 W), the yield of DHQ was not increased (decreased slightly in the range of error). Therefore, as a high extraction yield and a low energy consumption were required, a range of 150–250 W ultrasound power was selected for further optimization experiments.

#### Extraction cycles

**Figure 4:**
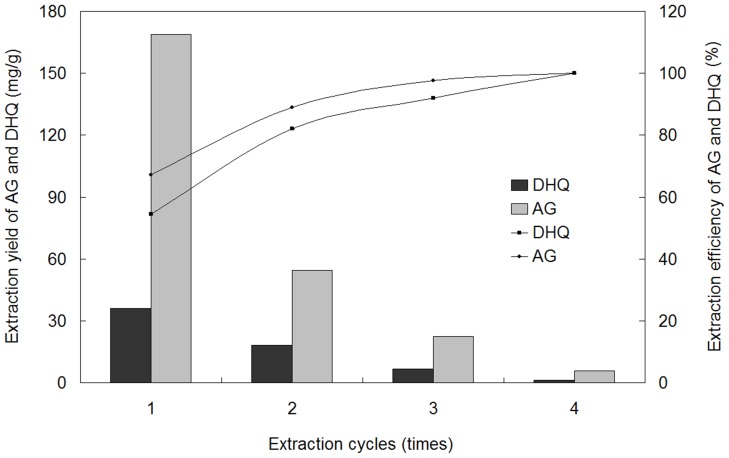
Effect of extraction cycles on the extraction yields of AG and DHQ.

The extraction conditions of soaking time, solid-liquid ratio, ultrasonic time, and ultrasonic power are 8 h, 1∶18 g/mL, 50 min and 200 W, respectively. To optimize extraction cycles, extractions were carried out four times. From Figure 4 (the original data, File S1), the total extraction yield of four cycles was defined as 100% (right-hand axis) and the extraction yields of AG and DHQ (left-hand axis) were compared with the total extraction yield. The extraction yield total for two cycles of AG reached 88.86% and the extraction yield total for two cycles of DHQ was 82.06% and the total for three cycles were more than 90% (97.71% for AG and 92.06% for DHQ). Considering these factors of time, solvent and energy consumption, an extraction process using three cycles seemed most reasonable.

### Optimization of UAE conditions

To further study the interactions between factors, we optimized the extraction time, ultrasound power and solid-liquid ratio, using the yields of AG and DHQ as the response values.

In Table 3 (the original data, File S2), the Model F-values of 64.21 and 15.42 implied that the model was significant. There is only a 0.01% chance that a “Model F-Value” this large could occur by chance. If values of “Probability >F” are less than 0.0500, this indicates that the model terms are significant. The Probability >F value of “model” were <0.0001 and 0.0008, respectively, It indicated that the experiment data were better fitting the model provided by RSM. In this case, For the yield of DHQ, *A* (the Probability >F value was <0.0001), *B* (the Probability >F value was <0.0001), *C* (the Probability >F value was <0.0001), *AC* (the Probability >F value was 0.0094), *BC* (the Probability >F value was 0.0094), *A^2^* (the Probability >F value was 0.0038), and *B^2^* (the Probability >F value was <0.0001) were significant model terms. And for the yield of AG, *A* (the Probability >F value was 0.0113), *B* (the Probability >F value was 0.0003), *C* (the Probability >F value was 0.0076), *A^2^* (the Probability >F value was 0.0032), *B^2^* (the Probability >F value was 0.0010), and *C^2^* (the Probability >F value was 0.0286). If values were greater than 0.1000 indicate that the model terms are not significant. If there are many insignificant model terms (not counting those required to support hierarchy), a reduction in the number of terms may improve the model.

**Table 3:** Test of significance for regression coefficient.^a^

Source	Df	*Y_1_* Sum of squares	*Y_2_* Sum of squares	*Y_1_* Mean square	*Y_2_* Mean square	*Y_1_* F-value	*Y_2_* F-value	*Y_1_* Pr>F	*Y_2_* Pr>F
* A*	1	0.36	172.98	0.36	172.98	18.13	11.63	0.0038	0.0113
* B*	1	5.61	673.45	5.61	673.45	281.57	45.27	<0.0001	0.0003
* C*	1	1.12	204.02	1.12	204.02	56.45	13.71	0.0001	0.0076
* AB*	1	2.500×10^−3^	41.60	2.500×10^−3^	41.60	0.13	2.80	0.7336	0.1384
* AC*	1	0.25	51.12	0.25	51.12	12.54	3.44	0.0094	0.1062
* BC*	1	0.25	1.56	0.25	1.56	12.54	0.11	0.0094	0.7553
* A^2^*	1	0.36	287.45	0.36	287.45	18.08	19.32	0.0038	0.0032
* B^2^*	1	3.35	430.58	3.35	430.58	168.30	28.95	<0.0001	0.0010
* C^2^*	1	0.019	112.22	0.019	112.22	0.96	7.54	0.3592	0.0286
Model b	9	11.52	2063.82	1.28	229.31	64.21	15.42	<0.0001	0.0008
Residual	7	0.14	104.13	0.020	14.88	——	——	——	——
Lack of fit	3	0.11	103.83	0.036	34.61	4.48	461.47	0.0908	<0.0001
Pure Error	4	0.032	0.30	8.000×10^−3^	0.075	——	——	——	——
Corr Total	16	11.66	2167.95	——	——	——	——	——	——
Linear	3	7.10	1050.44	2.37	350.15	6.75	4.07	0.0055	0.0304
Quadratic	3	3.92	919.09	1.31	306.36	65.5	20.59	<0.0001	0.0008
Cubic	3	0.11	103.83	0.036	34.61	4.48	461.47	0.0908	<0.0001

a The results were obtained with the Design Expert 7.0 software.

b *A* is extraction time (min), *B* is ultrasound power (W), *C* is solid-liquid ratio (w/v), and *Y_1_* is extraction yield of DHQ (mg/g), *Y_2_* is extraction yield of AG (mg/g).

Table 4 (the original data, File S2) shows that the standard deviations of the model were 0.14 and 3.86. The lower the CV value, the better the stability. In the present study, the CV values of *Y_1_* and *Y_2_* were low at 0.39% and 2.24%, respectively, indicating a high level of reliability for the experimental operation. The R^2^ values of 0.9880 and 0.9520 are in reasonable agreement with the adjusted R^2^ values of 0.9726 and 0.8902, indicating a high level of reliability for 90% of the experimental data. “Adequacy Precision” measures the signal-to-noise ratio where a ratio greater than 4 is desirable; the ratios of 26.785 and 9.795 indicated an adequate signal. This model can therefore be used to explore the design space to find the optimal values for extraction.

**Table 4:** Credibility analysis of the regression equations.

Index mark a	*Y_1_* is extraction yield of DHQ	*Y_2_* is extraction yield of AG
Std. Dev.	0.14	3.86
Mean	36.17	172.32
C.V. %	0.39	2.24
PRESS	1.77	1661.75
R-Squared	0.9880	0.9520
Adjust R-Squared	0.9726	0.8902
Predicted R-Squared	0.8481	0.2335
Adequacy Precision	26.785	9.795

a The results were obtained with the Design Expert 7.0 software.

The final extraction yields of DHQ (*Y_1_*) and AG (*Y_2_*) were found using RSM to be:

*Y_1_* = 36.76+0.36*A*+0.78*B*+0.54*C*-0.025*AB*+0.050*AC*-0.12*BC*-0.38*A* ^2^-0.81*B* ^2^-0.28*C* ^2^

*Y_2_* = 183.40+5.71*A*+9.55*B*+4.24*C*+3.22*AB*+4.45*AC*-0.13*BC*-9.70*A* ^2^-8.68*B* ^2^-5.85*C* ^2^

Where *A* is extraction time (min), *B* is ultrasound power (W), and *C* is solid-liquid ratio (g/mL), respectively.

**Figure 5:**
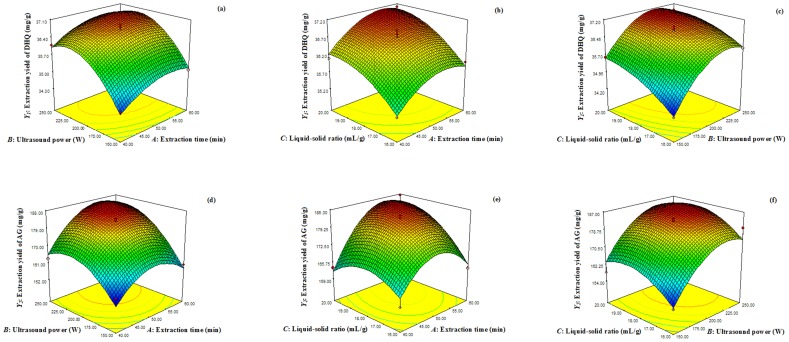
Response surfaces. (a) Response surface of the extraction yields of DHQ, *Y_1_* = f(A,B). (b) Response surface of the extraction yields of DHQ, *Y_1_* = f(A,C). (c) Response surface of the extraction yields of DHQ, *Y_1_* = f(B,C). (d) Response surface of the extraction yields of AG, *Y_2_* = f(A,B). (e) Response surface of the extraction yields of AG, *Y_2_* = f(A,C). (f) Response surface of the extraction yields of AG, *Y_2_* = f(B,C).

The response surfaces of the extraction yields of DHQ and AG were shown in Figure 5 (the original data, File S2).

As indicated by the equation above, the conditions for the optimal point predicted by the software were: ultrasound extraction 52.63 min at 200.15 W and 1∶18.32 solid-liquid ratios with extraction yields for AG and DHQ of 183.40 and 36.76 mg/g, respectively.

### Verification test under optimum conditions

The verification tests (the original data, File S1) were performed three times under the optimal conditions predicted by RSM. 30.0 g of dried sample was mixed with 40% ethanol (the solid-liquid ratio was 1∶18) soaked for 8 h, and then were ultrasound extracted at 200 W for 50 min. The actual extraction yields of AG and DHQ were 179.88±10.49 and 35.96±1.66 mg/g, respectively, with corresponding errors of about 1.92% and 2.18%.

### Comparison of different extraction methods

Water is the most common and inexpensive solvent, therefore pure water is always selected as the co-solvent in various extraction processes, with AG being soluble in water and DHQ in hot water. Table 5 (the original data, File S1) shows the extraction yields of AG and DHQ for different reference extraction methods (n = 3). The extraction yields of AG and DHQ using Soxhlet extraction for 12 h were the highest; 202.43±4.44 mg/g for AG and 69.3±2.95 mg/g for DHQ with 40%ethanol solution, and 223.43±4.88 mg/g for AG and 63.4±2.29 mg/g for DHQ with water. This extraction time was too long with higher energy consumption. The extraction yields of AG and DHQ using soaking at room temperature for 24 h were also high, reaching 164.22±4.28 mg/g for AG and 56.6±1.94 mg/g for DHQ, when the extraction solvent was 40%ethanol solution, and reaching 187.57±4.21 mg/g for AG and 40.0±2.74 mg/g for DHQ, when the extraction solvent was water. Although there was no energy consumption for this method, the extraction time was also too long and the extraction efficiency lower. Conversely, reflux extraction is a time-saving method, but consumes energy during the process of heating. The extraction yields of AG and DHQ with 40%ethanol solution reflux extraction for 2 h were 194.57±4.52 mg/g for AG and 46.5±1.71 mg/g for DHQ; the extraction yields of AG and DHQ with water reflux extraction for 2 h were 207.43±4.30 mg/g for AG and 32.4±1.32 mg/g for DHQ. The extraction yields of AG and DHQ using stirring extraction for 8 h with 40%ethanol solution at 50°C were 200.32±4.27 mg/g for AG and 60.7±2.15 mg/g for DHQ, and using stirring extraction for 8 h with water at 50°C were 218.42±5.31 mg/g for AG and 58.5±1.13 mg/g for DHQ, respectively. Compared with these extraction methods, the extraction yields of AG and DHQ using UAE were not the highest (183.40±4.26 mg/g for AG and 36.8±1.21 mg/g for DHQ with 40% ethanol, and 147.48±3.08 mg/g for AG and 17.6±0.86 mg/g for DHQ with), but it had the advantages of lower energy consumption and saving time. Moreover, when the solvent was 40% ethanol solution, it can obtain AG and DHQ simultaneously.

**Table 5:** Comparison of different extraction methods.

Item	Raw material	Extraction method	Extraction yield of AG (mg/g) (n = 3)	Extraction yield of DHQ (mg/g) (n = 3)
A	Wood flour	Soxhlet extraction 12 h with 40% ethanol solution	202.43±4.44	69.3±2.95
B	Wood flour	Soxhlet extraction 12 h with water	223.43±4.88	63.4±2.29
C	Wood chips	Soak 24 h with 40% ethanol solution at room temperature	164.22±4.28	56.6±1.94
D	Wood chips	Soak 24 h with water at room temperature	187.57±4.21	40.0±2.74
E	Wood chips	Reflux extraction 2 h with 40% ethanol solution	194.57±4.52	46.5±1.71
F	Wood chips	Reflux extraction 2 h with water	207.43±4.30	32.4±1.32
G	Wood chips	40% ethanol solution stirring extraction 8 h at 50°C	200.32±4.27	60.7±2.15
H	Wood chips	Water stirring extraction 8 h at 50°C	218.42±5.31	58.5±1.13
I	Wood chips	UAE 50 min with 40% ethanol solution	183.40±4.26	36.8±1.21
J	Wood chips	UAE 50 min with water	147.48±3.08	17.6±0.86

### Comparison of pulping processes and the physical properties of paper

Table 6 (the original data, File S1) shows that the pulp yield using UAE pretreatment was higher than that from untreated pulp and that the high-value active ingredients, AG and DHQ, could be obtained during this process. UAE not only increased the pulp yield but also created certain economic benefits. Moreover, the extraction of AG and DHQ using UAE pretreatment provides a convenient process for the subsequent pulp bleaching. The yield of HBSP was higher than that of KP, because the lignin removal during HBSP process is not as high compared with that during KP process. This can be explained by the kappa number of HBSP (77.91±0.06 for untreated and 77.01±0.06 for UAE-pretreated) being nearly three times higher than that for KP (27.30±0.13 for untreated and 26.83±0.08 for UAE-pretreated). However, compared with KP (the residual alkali value of untreated KP was 16.30±0.04, and that of UAE-pretreated KP was 16.55±0.06. It indicated that the alkali consumption was decreased through UAE pretreated), the natural advantage of the HBS pulping process is the recyclable solvent, which solves the problem of the high energy consumption for black liquor recovery and pollution of the environment during the process of alkaline pulping.

**Table 6:** Technical analysis of pulp.

Characters of pulping	Untreated KP	UAE-pretreated KP	Untreated HBSP	UAE-pretreated HBSP
Yield of pulp (%)	39.60	41.50	42.37	44.23
Kappa number (n = 3)	27.30±0.13	26.83±0.08	77.91±0.06	77.01±0.06
Residual alkali value (g·L^−1^) (n = 3)	16.30±0.04	16.55±0.06	——	——

Table 7 (the original data, File S1) shows that many physical properties paper were improved by UAE-pretreatment. The basis weight of paper was lower (85.67±0.64 and 82.48±0.55 g·cm^−2^ for paper from untreated KP and HBSP; 79.94±0.32 and 80.25±0.55 g·cm^−2^ for paper from UAE-pretreated KP and HBSP, respectively). The apparent densities of paper were nearly the same (0.3273±0.0016 and 0.3085±0.0005 g·cm^−3^ for paper from untreated KP and HBSP; 0.3272±0.0034 and 0.3054±0.0014 g·cm^−3^ for paper from UAE-pretreated KP and HBSP, respectively). The tensile strengths were slightly higher (38.69±0.07 and 37.25±0.07 N·m·g^−1^ for paper from untreated KP and HBSP; 38.75±0.11 and 37.90±0.13 N·m·g^−1^ for paper from UAE-pretreated KP and HBSP, respectively). The tearing strengths were higher (14.25±0.08 and 14.12±0.06 mN·m^2^·g^−1^ for paper of untreated KP and HBSP; 16.88±0.08 and 16.22±0.06 mN·m^2^·g^−1^ for paper of pretreated KP and HBSP, respectively). The bursting strengths were slightly higher (2.17±0.04 and 1.87±0.06 Kpa·m^2^·g^−1^ for paper from untreated KP and HBSP; 2.25±0.04 and 1.93±0.05 Kpa·m^2^·g^−1^ for paper from UAE-pretreated KP and HBSP, respectively). The folding strengths (8±1 and 6±1 times for paper from untreated KP and HBSP; 11±1 and 6±0 times for paper from UAE-pretreated KP and HBSP, respectively) of paper were higher. The reason for these changes is that during the process of UAE-pretreatment, compounds such as DHQ, the water-soluble AG, tannin and polyphenols, which are harmful to the pulping and papermaking processes, are removed. This improves the reaction efficiency of pulping and also the quality of the resulting paper.

**Table 7:** Physical properties of paper.

Physical properties	Paper of untreated KP	Paper of UAE-pretreated KP	Paper of untreated HBSP	Paper of UAE-pretreated HBSP
Weight (g) (n = 3)	2.69±0.02	2.51±0.01	2.59±0.02	2.52±0.02
Basis weight (g·cm^−2^) (n = 3)	85.67±0.64	79.94±0.32	82.48±0.55	80.25±0.55
Apparent density (g·cm^−3^) (n = 3)	0.3273±0.0016	0.3272±0.0034	0.3085±0.0005	0.3054±0.0014
Tensile strength (N·m·g^−1^) (n = 6)	38.69±0.07	38.75±0.11	37.25±0.07	37.90±0.13
Tearing strength (mN·m^2^·g^−1^) (n = 6)	14.25±0.08	16.88±0.08	14.12±0.06	16.22±0.06
Bursting strength (Kpa·m^2^·g^−1^) (n = 6)	2.17±0.04	2.25±0.04	1.87±0.06	1.93±0.05
Folding strength (times) (n = 6)	8±1	11±1	6±1	6±0

## Conclusions

In summary, ultrasound-assisted extraction (UAE) was used for extracting arabinogalactan (AG) and dihydroquercetin (DHQ) simultaneously from *Larix gmelinii* wood, as a pretreatment for pulping and papermaking. Compared with untreated wood chips, the pulping characteristics and physical properties of the resulting paper were improved. The extraction parameters were optimized by Response Surface Methodology. The optimum conditions were three extraction cycles using 40% ethanol with 50 min for each cycle, ultrasound power at 200 W and a solid-liquid ratio of 1∶18, leading to extraction yields for AG and DHQ of 179.88±10.49 and 35.96±1.66 mg/g, respectively. The UAE pretreatment of wood chips not only improved the pulping characteristics and physical properties of paper, but also obtained substantial yields of the active compounds, AG and DHQ. The data from the present study will contribute to promoting the extraction of these valuable active compounds from larch wood.

## Supporting Information

File S1 **Original data of** **Table 1** **,** **Table 2** **,** **Table 5** **,** **Table 6** **,** **Table 7** **, and** **Figure 2** **,** **Figure 3** **,** **Figure 4** **, and verification test.**
(XLS)

File S2 **Original data of** **Table 3** **,** **Table 4** **, and** **Figure 5** **.**
(DX7)
